# Synthesis and characterization of WS_2_/graphene/SiC van der Waals heterostructures via WO_3−x_ thin film sulfurization

**DOI:** 10.1038/s41598-020-74024-w

**Published:** 2020-10-15

**Authors:** Jonathan Bradford, Mahnaz Shafiei, Jennifer MacLeod, Nunzio Motta

**Affiliations:** 1 School of Chemistry and Physics, Queensland University of Technology (QUT), Brisbane, QLD Australia; 2 School of Physics and Astronomy, University of Nottingham, Nottingham, NG7 2RD UK; 3 Faculty of Science, Engineering and Technology, Swinburne University of Technology, Hawthorn, VIC Australia; 4 Institute for Future Environments, Queensland University of Technology (QUT), Brisbane, QLD Australia; 5 Centre for Materials Science, Queensland University of Technology (QUT), Brisbane, QLD Australia

**Keywords:** Electronic properties and materials, Synthesis and processing, Two-dimensional materials, Graphene, Scanning probe microscopy, Raman spectroscopy, Characterization and analytical techniques

## Abstract

Van der Waals heterostructures of monolayer transition metal dichalcogenides (TMDs) and graphene have attracted keen scientific interest due to the complementary properties of the materials, which have wide reaching technological applications. Direct growth of uniform, large area TMDs on graphene substrates by chemical vapor deposition (CVD) is limited by slow lateral growth rates, which result in a tendency for non-uniform multilayer growth. In this work, monolayer and few-layer WS_2_ was grown on epitaxial graphene on SiC by sulfurization of WO_3**−**x_ thin films deposited directly onto the substrate. Using this method, WS_2_ growth was achieved at temperatures as low as 700 °C – significantly less than the temperature required for conventional CVD. Achieving long-range uniformity remains a challenge, but this process could provide a route to synthesize a broad range of TMD/graphene van der Waals heterostructures with novel properties and functionality not accessible by conventional CVD growth.

## Introduction

Transition metal dichalcogenides (TMDs) are a family of two-dimensional (2D) materials that exhibit an interesting range of layer dependent properties. Semiconducting TMDs such as MoX_2_ and WX_2_ (where X represents S or Se) exhibit a layer-dependent bandgap with an indirect to direct transition when isolated as a monolayer^1,2^. Furthermore, owing to broken inversion symmetry, monolayer TMDs also possess strongly spin-split valence states, where the splitting exhibits opposite signs at *K* and *K’* valleys enabling optically induced spin and valley polarization^3–5^. In recent years, research has turned towards creating heterostructures of TMDs and graphene due to the appeal of capitalizing on the complementary properties of the two materials^6,7^. For example, the exceptional carrier mobility of graphene complements the direct optical bandgap of monolayer TMDs such as molybdenum disulfide (MoS_2_) and tungsten disulfide (WS_2_)^2,8^. In addition, spin polarization in TMDs is complemented by long spin lifetimes in graphene^9,10^. When combined in a heterostructure these two materials can deliver outstanding performance in a range of applications including optoelectronics^11–13^, spintronics and valleytronics^14–16^, sensing^17,18^, and energy storage^19,20^.

To date, chemical vapor deposition (CVD) of TMDs has been used to successfully to produce monolayers of MoS_2_, WS_2_, MoSe_2_ and WSe_2_ at the wafer scale on substrates such as SiO_2_/Si and sapphire^21,22^. Despite these successes there are still some limitations in extending this process to synthesizing large area monolayers on van der Waals substrates such as graphene. Studies of CVD growth of TMDs on graphene typically report limited monolayer domain size before the onset of multilayer growth^23–28^. A fundamental limitation of CVD growth of TMDs on graphene compared to traditional substrates (i.e. SiO_2_/Si or sapphire) is the weak adsorption of the precursors on the surface, which results in slow lateral growth rates and a tendency for multilayers to form^29^. Specifically, the adsorption energy of chalcogen adatoms is approximately half that of metal adatoms, which in turn is more than an order of magnitude larger than the diffusion energy barrier. Thus, at elevated growth temperatures, the chalcogen-deficient environment and large diffusion of metal adatoms results in growth of multilayer pyramids^29^. The upper limit of monolayer domain size for TMDs grown on graphene by CVD is currently in the 10 μm range^28^. Therefore, there is impetus to develop alternative approaches to synthesizing TMDs on graphene substrates.

Previous studies have demonstrated direct sulfurization of the metal precursor as an effective route to producing large-area TMDs on SiO_2_/Si^30–32^. This has the advantage of allowing a control of the layer thickness and morphology directly according to the thickness of the precursor. For instance, it has previously been demonstrated that the thickness of a metal precursor layer can be used to grow TMD layers with the basal plane oriented either horizontally or vertically with respect to the substrate^31,33^. In this work, we have produced WS_2_/graphene/SiC van der Waals heterostructures by depositing WO_3**−**x_ thin films directly onto epitaxial graphene/SiC substrates followed by sulfurization to produce WS_2_/graphene/SiC van der Waals heterostructures. We have utilized scanning tunneling microscopy (STM), atomic force microscopy (AFM), photoemission spectroscopy and Raman spectroscopy to investigate the surface morphology and composition of the WS_2_ layers and have presented the electronic band alignment of the van der Waals heterostructure.

## Results

### Sulfurization of WO_3−x_ Thin Films

**Figure 1:**
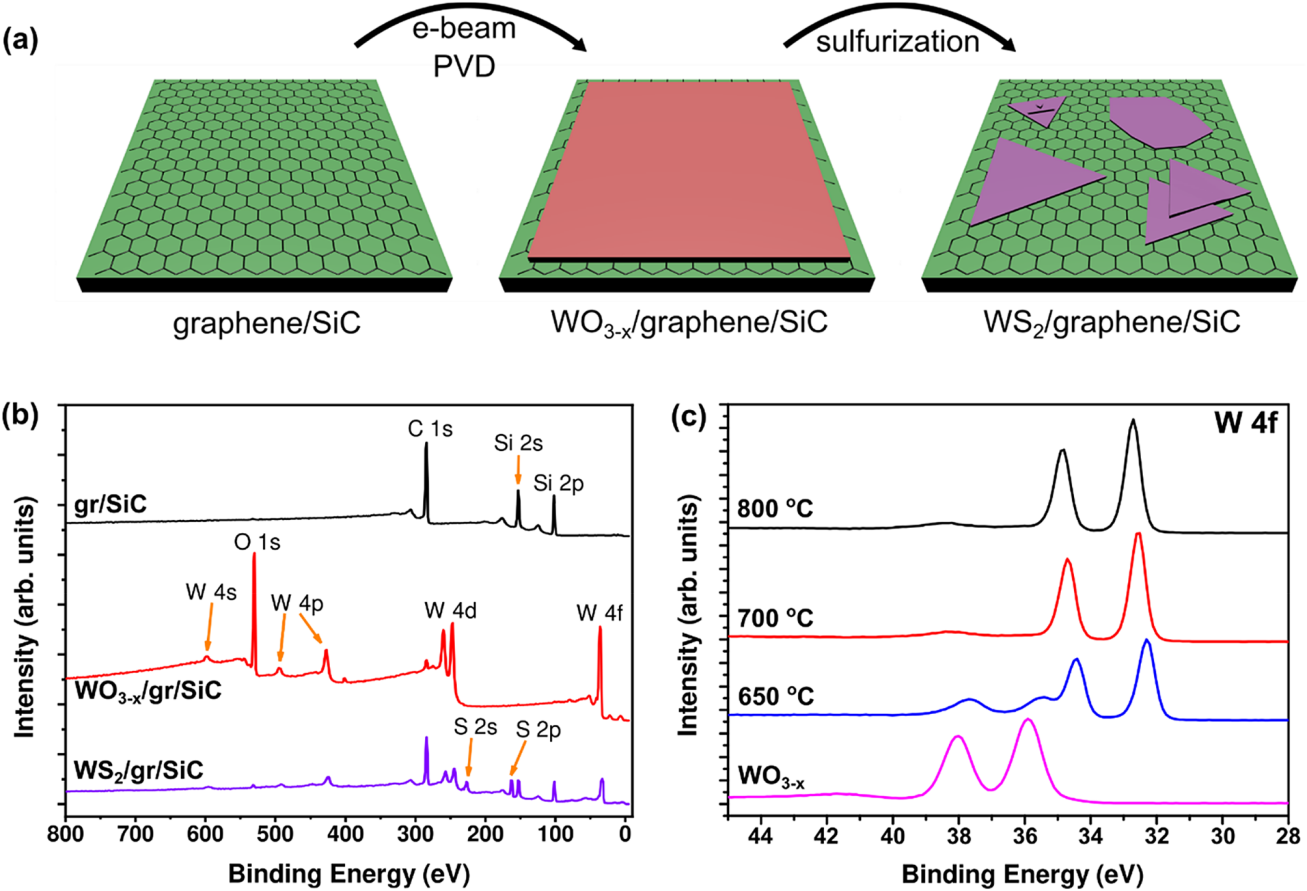
(**a**) Schematic of the WS_2_ growth process on epitaxial graphene/SiC(0001); (**b**) Evolution of the XPS survey spectra of at each stage of the WS_2_ growth procedure; (**c**) Comparison of the W 4f core level spectra at different sulfurization temperatures.

WS_2_ layers were grown following the procedure shown schematically in Fig. 1(a), and detailed in the Experimental Section. After growing the graphene layer in UHV, a thin layer of tungsten oxide was deposited by electron-beam physical vapor deposition (e-beam PVD) to provide a tungsten source for WS_2_ growth. The sample was then characterized by XPS, AFM and Raman spectroscopy to determine the surface chemistry and morphology (data is presented in the Supporting Information). Figure S1 shows data from a sample with a nominal tungsten oxide thickness of 10 nm. Elemental quanification based on the XPS data (Figure S1(a),(b), Supporting Information) indicates that the O:W ratio in the tungsten oxide layer is 2.5 ± 0.3. AFM allows a direct measurement of the WO_3**−**x_ layer thickness, which was found to be 10.8 nm (Figure S1(c),(d), Supporting Information). The Raman spectrum of the layer (Figure S2, Supporting Information) shows no features that could be attributed to WO_3_, which may be due to the small layer thickness, or poor crystallinity of the WO_3**−**x_.

Evolution of the surface chemistry during the WS_2_ growth process can be observed in the XPS surveys shown in Fig. 1(b). A similar comparison of the high-resolution spectra can be found in Figure S3 (Supporting Information). The bare epitaxial graphene spectrum (black) shows only carbon and silicon peaks, and is free of oxygen, indicating a clean graphene surface. After e-beam deposition of WO_3**−**x_ (red spectrum) the silicon peaks disappear due to the thickness of the WO_3**−**x_ plus graphene layers exceeding the sampling depth of elastically scattered photoelectrons. Inspection of the high-resolution C 1 s core level indicates that the carbon content is mostly graphene with a small amount of adventitious carbon on the surface. As mentioned previously, quantification of the W 4f and O 1 s core levels points to a sub-stoichiometric oxide layer WO_3**−**x_ where x = 0.5 ± 0.3. After sulfurization of the WO_3**−**x_ layer the XPS survey (purple) shows that the oxygen is almost completely removed, and the carbon and silicon peak intensity re-emerge. These trends are indicative of partial evaporation of the oxide layer during sulfurization to produce a WS_2_ thickness of only a few layers as observed by STM (discussed later). It is noted that calculation of the number of WS_2_ layers from the W 4f and S 2p core level intensities is not straightforward due to the inhomogeneity of the number of layers and coverage of WS_2_.

To determine the lower temperature limit for WS_2_ growth, different samples of WO_3**−**x_/graphene/SiC were sulfurized at different temperatures under otherwise identical conditions, and the chemical composition was monitored by XPS. Figure 1(c) shows the W 4f. core level spectrum at sulfurization temperatures of 800 °C, 700 °C and 650 °C, as well as the spectrum for WO_3**−**x_ before sulfurization. The samples sulfurized at 800 °C and 700 °C contain a single chemical state with the 4f_7/2_ peak at approximately 32.7 eV with a spin–orbit peak separation of 2.1 eV corresponding to fully sulfurized WS_2_. There is a shift in the peak positions to higher binding energies as the sulfurization temperature increases. The binding energy of W 4f electrons in WS_2_ has previously been shown to be sensitive to the stoichiometry^34^, however the S:W ratios for the samples sulfurized at 800 °C and 700 °C are 2.1 ± 0.1 and 2.2 ± 0.3, respectively, and cannot be differentiated within the bounds of uncertainty. In addition to the WS_2_ contribution, a second component is present in the sample sulfurized at 650 °C at 35.5/37.6 eV which is a slightly lower binding energy compared to the WO_3**−**x_ reference spectrum.

**Figure 2:**
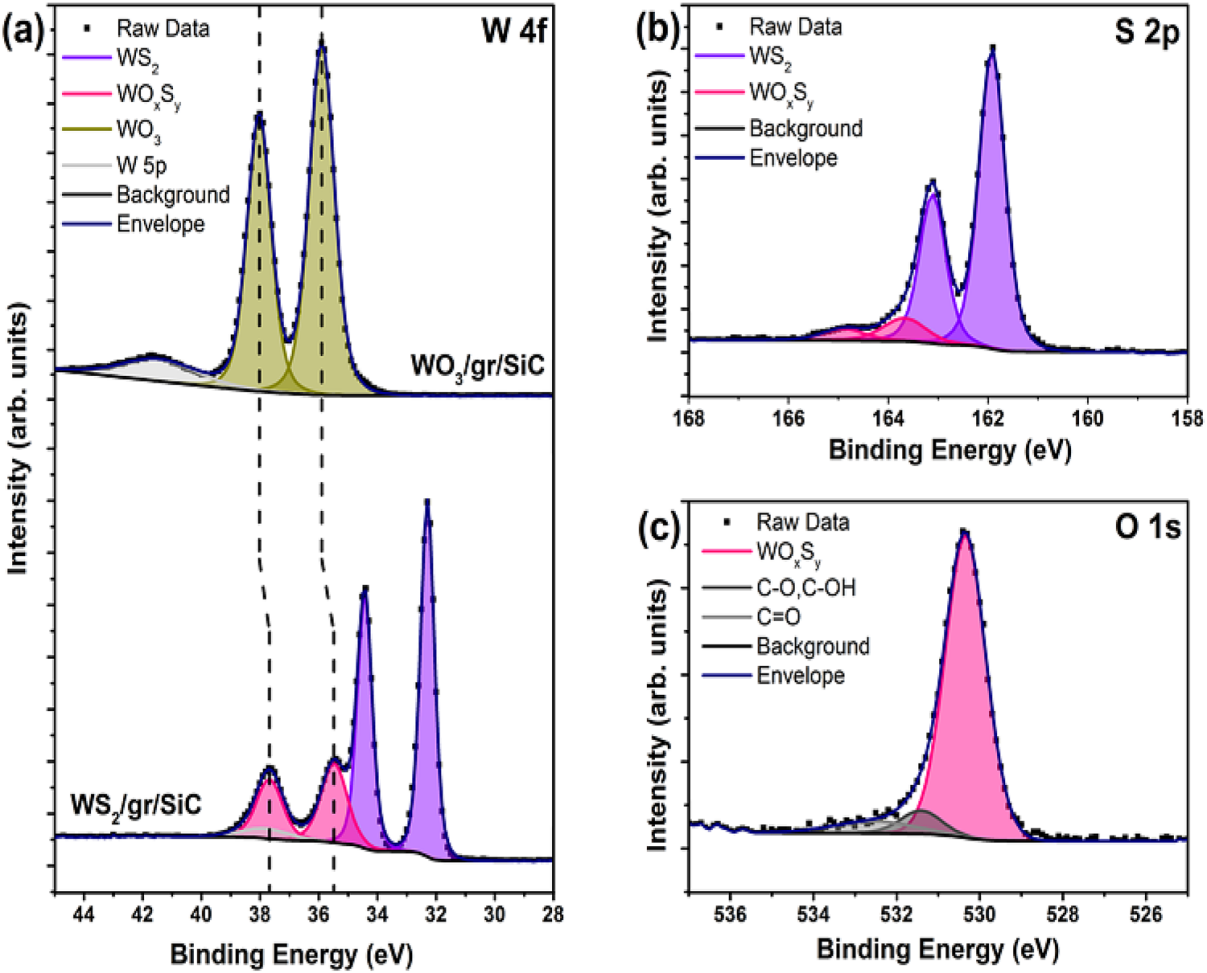
High resolution XPS core level spectra of partially sulfurized WO_x_S_y_ grown at a sulfurization temperature of 650 °C. (**a**) Peak deconvolution of the W 4f core level before (top) and after (bottom) sulfurization; (**b**, **c**) S 2p and O 1 s core level spectra, respectively, after sulfurization.

Figure 2 shows the peak deconvolution of the core level spectra from the sample sulfurized at 650 °C. The higher binding energy component observed in the W 4f core level (Fig. 2(a), bottom) is also present in the S 2p (Fig. 2(b)) at 163.7 eV and 164.9 eV for S 2p_3/2_ and S 2p_1/2_, respectively, and at 530.4 eV in the O 1 s core level. We therefore ascribe the peak to a tungsten oxysulfide compound (WO_x_S_y_)^35–37^. Based on the peak area ratios from the respective core levels, the composition of the WO_x_S_y_ compound was determined to be $x = 2.03 \pm 0.37$ and $y = 0.72 \pm 0.09$. The formation of WO_x_S_y_ is believed to occur in the initial stages of WO_3_ sulfurization where the oxide is reduced followed by incorporation of sulfur to form a tungsten oxysulfide intermediate. Previous work has demonstrated that complete sulfurization of crystalline WO_3_ can occur at temperatures as low as 400°C^37^. The incomplete sulfurization observed in this work is likely due to the sulfur feedstock becoming fully depleted before the reaction is complete. It is possible that sulfurization using a H_2_S sulfur precursor may allow longer sulfurization times at lower temperatures^37^.

### WS_2_ Layer Characterization

**Figure 3:**
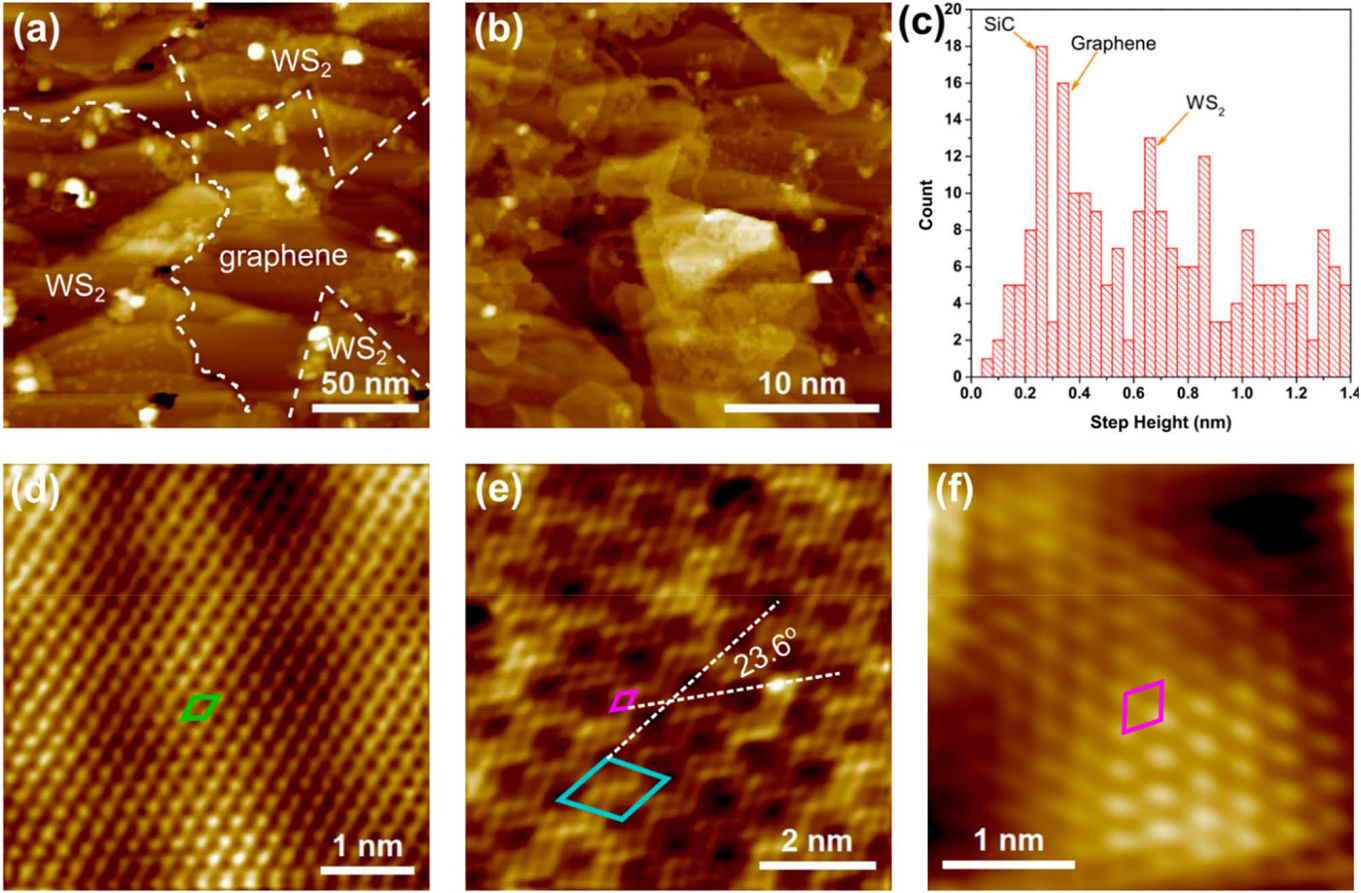
(**a**) STM image of monolayer WS_2_ on epitaxial graphene. The WS_2_ is outlined by the dashed white line (U = −1.50 V, I = 1.0 nA); (**b**) Multilayer WS_2_ on a different region of the sample (U = −1.50 V, I = 0.7 nA); (**c**) Distribution of step heights measured on STM images of WS_2_/graphene/SiC; (**d**) Atomic resolution image of a graphene region after WS_2_ growth. The unit cell is marked by the green rhombus (U = −0.08 V, I = 0.1 nA); (**e**) Atomic resolution STM image of monolayer WS_2_ on epitaxial graphene. The unit cell of the WS_2_ lattice is drawn in magenta, and the unit cell of the moiré pattern is drawn in blue (U = −1.57 V, I = 0.1 nA); and (**f**) STM image of multilayer WS_2_ on graphene showing the WS_2_ lattice without a moiré pattern (U = −1.67; V, I = 1.2 nA); the unit cell is marked in magenta.

Next, we present a detailed surface characterization of the sample sulfurized at 800 °C which produced the thinnest WS_2_ layers. The surface morphology was studied by STM as shown in Fig. 3(a) and 3(b). These images show two regions of the surface with different coverages of WS_2_. Figure 3a shows a region containing mostly monolayer coverage of WS_2_ in the areas outlined by dashed lines. It contains both triangular domains (bottom-right and top-right), as is common for TMDs grown by CVD, and a larger irregularly shaped domain (left). A different region of the sample is shown Fig. 3b with disordered WS_2_ multilayers highlighting the inhomogeneity of the WS_2_ morphology across the sample. Additional STM data and discussion on the assignment of the WS_2_ regions can be found in Figure S4, Supporting Information. A histogram of the step heights extracted from multiple images of the WS_2_/graphene/SiC surface (all measured at a sample bias of −1.5 V), shown in Fig. 3(c), exhibits prominently recurring step heights at 0.25 nm, 0.35 nm and ~ 0.68 nm corresponding to the layer thicknesses of SiC, graphene and WS_2_, respectively^38,39^.

Atomic resolution images of an exposed graphene region, monolayer WS_2_, and multilayer WS_2_ are shown in Fig. 3(d), (e), and (f), respectively. The graphene lattice (unit cell indicated in green) shows only three of the six carbon atoms in graphene indicating that the region contains at least two graphene layers. The AB stacking of multiple graphene layers (similar to that in bulk graphite) breaks the symmetry of the graphene sublattices causing the altered appearance in STM^38,40^. The atomically resolved image of monolayer WS_2_ in Fig. 3(e) shows a small periodicity of 3.10 ± 0.13 Å (unit cell drawn in magenta) corresponding to WS_2_ as well as a moiré pattern with 10.28 ± 0.26 Å periodicity indicated by the blue unit cell. This corresponds to a (4 × 4) moiré structure of graphene, and the angle between the moiré pattern and WS_2_ lattice is 23.6°. Moiré superstructures are known to occur in TMDs grown on graphene: they are usually associated with small rotational misalignment between the TMD and graphene lattices^28^. For example, Ugeda et al*.* observed in MoSe_2_ grown on epitaxial graphene that a slight rotation of 3° of the MoSe_2_ monolayer with respect to graphene produces a 9.87 Å moiré superstructure, which accommodates four graphene and approximately three MoSe_2_ unit cells^41^. Unstrained graphene and WS_2_ are non-commensurate, however it has previously been shown that the residual strain in epitaxial graphene can improve commensurability between graphene and MoS_2_^23^. It has been demonstrated for both MoS_2_ and WS_2_ that the TMD preferred orientation with respect to graphene is either 0° or 30° with fluctuations of a few degrees^25,27,39^. Chen et al*.* related the misalignment of the moiré lattice ($\phi$) with the interlayer twist angle ($\theta$) of two non-identical hexagonal lattices using Eq. (1)^42^:

1 $$\theta = \tan^{ - 1} \left( {\frac{\sin \phi }{{\frac{D}{{a_{top} }} + \cos \phi }}} \right)$$

where $D$ is the moiré periodicity and $a_{top}$ is the lattice constant of the top layer. Based on the values obtained by STM for the WS_2_ overlayer on epitaxial graphene we can extract an interlayer twist angle $\theta = 5.4^{ \circ }$. Moiré patterns were only observed in regions of monolayer WS_2_ and in multilayer regions as shown in Fig. 3f, the WS_2_ lattice is observed without any moiré pattern.

**Figure 4:**
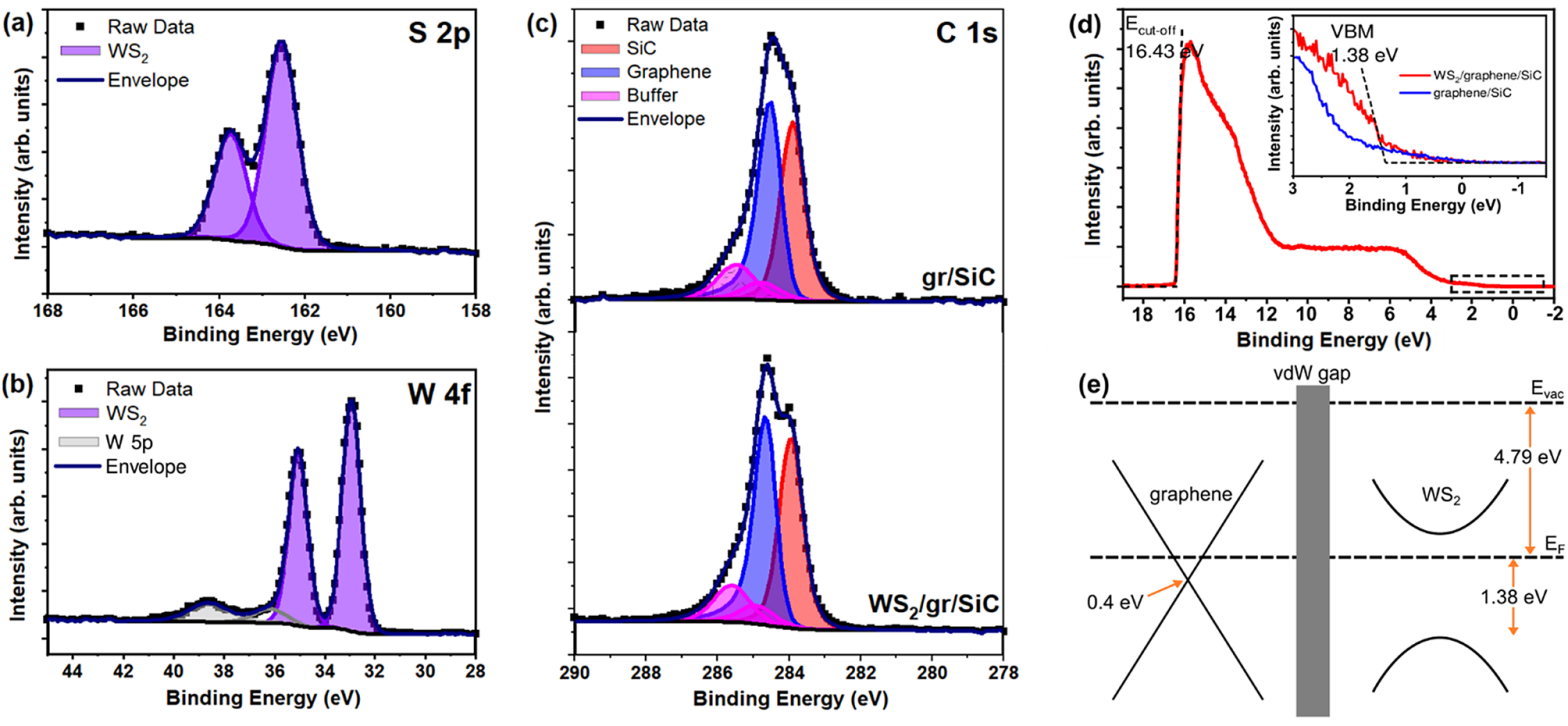
Photoemission spectroscopy of WS_2_/graphene/SiC. (**a**, **b**) XPS S 2p and W 4f core levels, respectively, after WS_2_ synthesis, and (**c**) shows the C 1 s core level of bare epitaxial graphene (top) and WS_2_/graphene/SiC (bottom); (**d**) Valence band spectrum of WS_2_/graphene/SiC. The inset shows a higher magnification of the spectrum near the Fermi level, with the blue spectrum for the bare graphene sample included for comparison; and (**e**) Proposed band alignment of the WS_2_/graphene van der Waals heterostructure.

Figure 4(a) and (b) show the S 2p and W 4f core level spectra after sulfurization of the WO_3**−**x_ layer. The S 2p shows only a single doublet at 162.5 eV and 163.7 eV, which corresponds well with the binding energy of sulfur in WS_2_. Similarly, the W 4f core level shows only a single chemical state corresponding to WS_2_ with W 4f_7/2_ and W 4f_5/2_ at 32.9 eV and 35.1 eV, respectively^27,43^. We note that nearby the W 5p_3/2_ peak (shaded in grey), an additional peak is required for a good fit to the experimental data. It is likely that this peak is required only to compensate for an imperfect background subtraction. It cannot correspond to residual WO_3**−**x_ since there is minimal oxygen left of the surface after sulfurization, and the high-resolution O 1 s core level shows only a single component at 531.9 eV corresponding to C-O_x_ species rather than metal oxide (Figure S3, Supporting Information). W 4f. and S 2p core levels reveals the ratio of S:W to be 1.97 ± 0.05 indicating that the WS_2_ is stochiometric. Figure 4(c) shows the C 1 s core level spectrum before (top) and after (bottom) WS_2_ synthesis. In both cases peaks corresponding to the SiC substrate, graphene layer and buffer layer are present in the same ratio indicating that graphene is unaffected during the growth process. The change in the envelope shape is caused by a slight shift of the sp^2^ C–C component by −0.1 eV after WS_2_ growth, and may be caused by band bending of the C 1 s core level in the heterostructure^27^. Notably there are no components corresponding to C-W or C-S bonds, which means the WS_2_ is a true van der Waals layer with no chemical bonds or strong chemical interaction at the interface.

Valence states in WS_2_ were probed by UPS and are shown in Fig. 4(d). From the secondary cut-off energy ($E_{cut - off} = 16.34$ eV) we extract the work function of $\phi = h\nu - E_{cut - off} = 4.79$ eV, where $h\nu$ is the photon energy of the HeI UV source (21.22 eV). From the valence band spectrum, the valence band maximum (VBM) can also be extracted. The inset of Fig. 4(d) shows the region close to the Fermi level in more detail. The red line is the measurement acquired from WS_2_/graphene/SiC, and the blue line shows the region for the bare epitaxial graphene before WS_2_ synthesis. The spectra show the valence band edge of WS_2_ superimposed on the continuum of states down to the Fermi level contributed by the graphene layer. The position of VBM for WS_2_ is located at 1.38 eV below the Fermi level and is consistent with the observed VBM for sulfur-deficient edges in monolayer WS_2_ (1.35 eV)^44^. Previous reports have placed the VBM for WS_2_ on graphene/SiC between 1.13 eV and 1.84 eV below the Fermi level^27,28,43^. Differences in the VBM can arise due to the intrinsic charge density, but it is also noted that the VBM shifts depending on the number of layers^28,45,46^. Due to the spot size of the UV source it is likely that the measurement is sampling many WS_2_ domains which may have different numbers of layers.

Based on the UPS measurement we propose the heterostructure band alignment shown in Fig. 4(e). The position of graphene’s Dirac cone, depicted on the left, has the Dirac point 0.3–0.4 eV below the Fermi level, as has been reported in multiple works^47,48^, noting that the band structure is not significantly perturbed after WS_2_ growth by CVD^27^. The position of the WS_2_ conduction band minimum (CBM) is approximately 0.4 eV above the Fermi level and is based on time- and angle-resolved photoemission spectroscopy (TR-ARPES) measurements of monolayer WS_2_^49^. The CBM of TMDs is dominated by *d*-states of the transition metal atom sandwiched between chalcogen atoms and is therefore less sensitive to number of layers than the VBM. Based on this measurement, the resulting WS_2_ bandgap is approximately 1.78 eV and is in good agreement with the bandgap measured by scanning tunneling spectroscopy (STS) of 3L MoS_2_ which is known to have a very similar electronic band structure^1,2,24^.

**Figure 5:**
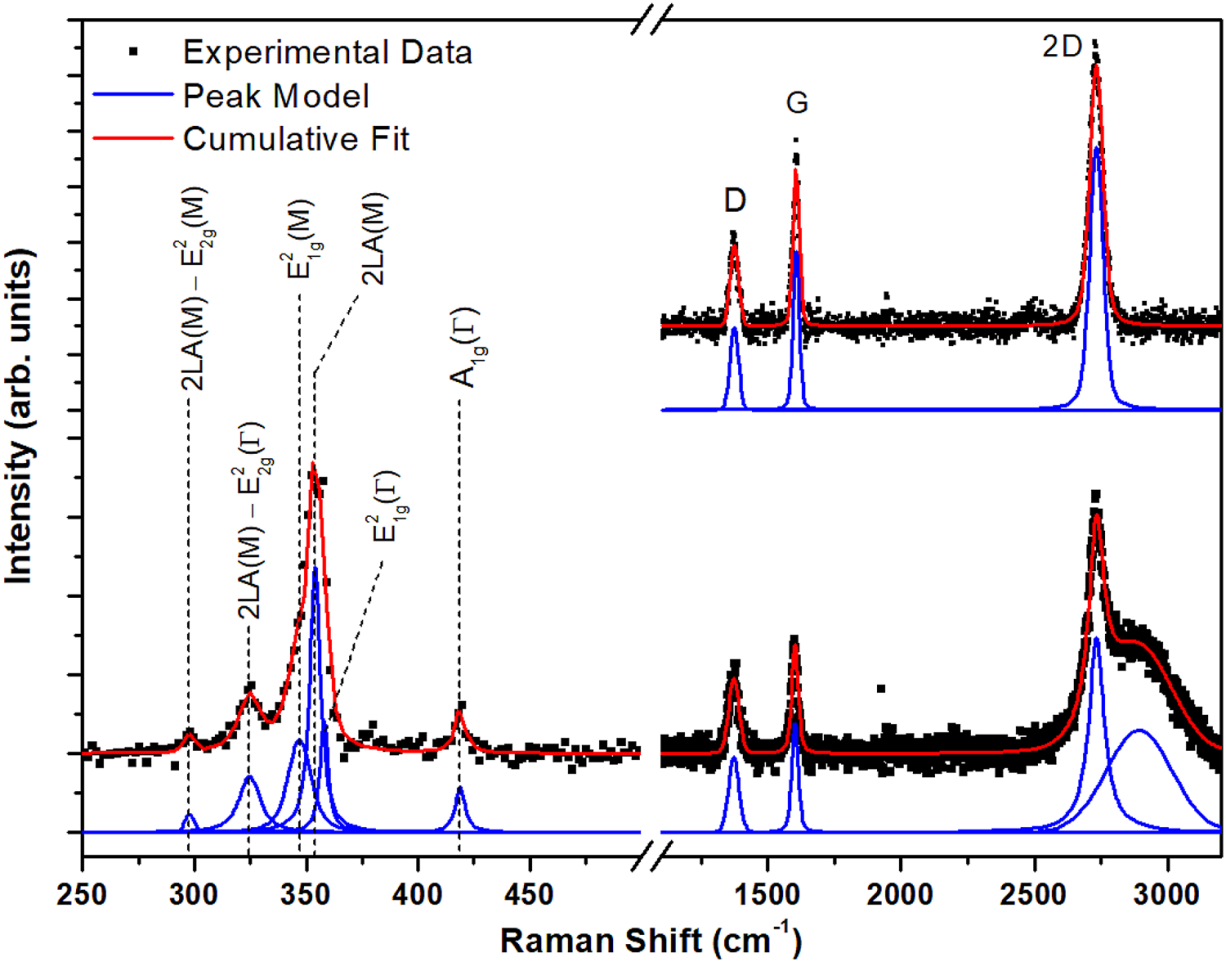
Raman spectrum of WS_2_ grown on epitaxial graphene/SiC. The spectrum of the bare epitaxial graphene is shown in the top right.

The Raman spectrum of WS_2_/graphene/SiC is shown in Fig. 5 with the spectrum of epitaxial graphene before WS_2_ synthesis presented in the top right. The 532 nm excitation used to acquire the spectrum is in resonance with the B exciton of WS_2_ which produces a series of combination modes in addition the in-plane and out-of-plane vibrational modes^43,50–52^. The A_1g_(G) peak at 419 cm^−1^ corresponds to an out-of-plane vibration of S atoms in the S-W-S stack. The large feature at ~ 350 cm^−1^ can be deconvolved into components corresponding to the E_2g_^1^(G) in-plane vibration of W and S atoms at 358 cm^−1^, the 2LA(M) overtone of longitudinal acoustic phonons at 354 cm^−1^, and E_2g_^1^(M) mode at 347 cm^−1^. In addition, peaks at 325 cm^−1^ and 298 cm^−1^ correspond to the 2LA(M)-E_2g_^2^(G) and 2LA(M)-2E_2g_^2^(G) combination modes, respectively. The A_1g_(G) and E_2g_^1^(G) peak separation has been established as a useful parameter in determining the number of WS_2_ layers^51^. In our case, the peak separation is 61 ± 2 cm^−1^ which is indicative of 1–3 WS_2_ layers and is consistent with local observations of the number of WS_2_ layers by STM.

The graphene Raman spectrum consists of three modes: D, G and 2D, which are positioned at 1374 cm^−1^, 1606 cm^−1^ and 2732 cm^−1^, respectively. The broad band at 2893 cm^−1^ arises due to photoluminescence (PL) from WS_2_^13^. In comparison to the Raman spectrum for epitaxial graphene (shown in the top right of Fig. 5), the intensity of ratio of the D and G bands (I_D_/I_G_) has increased from 0.64 ± 0.03 to 0.95 ± 0.08. This result suggests that the WS_2_ growth process has induced some defects in the graphene layer. The G and 2D bands of graphene are also observed to shift after WS_2_ synthesis. The G band frequency becomes redshifted by −4.8 ± 1.1 cm^−1^, and the 2D peak may slightly blueshifted although the shift is within the bounds of uncertainty (${\Delta }\overline{\nu }_{2D} = 0.6 \pm 0.6$ cm^−1^). Noting that a laser power of < 3.5 mW was used to prevent local heating and strain induced by the laser, the origin of the peak shift is due to charge transfer between graphene and WS_2_. This is consistent with laser-induced hole-doping of the graphene layer since epitaxial graphene is n-type (see Fig. 4e): a redshift of the G peak suggests a change in carrier concentration closer to charge neutrality, and the shift in the 2D peak position is minimal for low electron concentrations^53^. Since the laser photon energy (2.33 eV) exceeds the bandgap of WS_2_, it will generate electron–hole pairs which are then separated at the interface between graphene and WS_2_ layers^54^. Shifts in the G and 2D indicate that the electrons remain in WS_2_ and holes move into graphene thereby reducing the electron concentration in the graphene layer.

## Discussion

The main result of this study is the growth of WS_2_ layers at temperatures as low as 700 °C, with an optimal growth temperature of 800 °C, by utilizing sulfurization of a pre-deposited WO_3**−**x_ layer. At this temperature we were able to produce WS_2_ layers down to monolayer thickness. Though we have locally identified WS_2_ with a slight misalignment of 5.4° with respect to the graphene lattice, on a macroscopic scale there is no preferred orientation as determined by low-energy electron diffraction (Figure S5, Supporting Information). This may be due to the reduced growth temperature compared to the CVD growth used in other studies where there is a preferred orientation.

Direct sulfurization of WO_3_ thin films to grow WS_2_ layers on epitaxial graphene presents two advantages over conventional CVD: (1) Sufficient supply of the tungsten precursor is guaranteed during growth, allowing for higher coverages of thin WS_2_ layers rather than thicker multilayer structures; and (2) the metal oxide precursor no longer needs to be volatilized thus allowing lower growth temperatures compared to CVD. As a comparison to the sulfurization method we also used CVD only to grow WS_2_ from WO_3_ and sulfur powder precursors following the procedure outlined in the Supporting Information. In this case, in order to produce WS_2_ layers a growth temperature of 1100 °C was required (see Figure S6, Supporting Information). Typically WS_2_ synthesis by CVD is more challenging than MoS_2_ synthesis due to the difficulty in volatilizing the WO_3_ precursor, which requires temperatures of exceeding 900 °C^13,28,55,56^. In comparison to WS_2_ grown by thin film sulfurization which results in numerous grains of thin (1–3 layers) WS_2_, conventional CVD resulted in the formation of isolated, thick multilayer (> 10 layers) WS_2_ crystals.

Due to the low diffusion barrier of the metal presursors on graphene, and the low adsorption energy of chalcogen species, slow growth rates over long time periods are required to produce extended TMD monolayers on epitaxial graphene^28,29^. This presents a fundamental limitation of the CVD process since the metal oxide precursor cannot be sufficiently isolated from the chalcogen source, and the supply is therefore limited due to sulfurization of the source material. This matter becomes a significant problem in extending the CVD procedure to synthesize TMDs beyond the commonly studied molybdenum and tungsten disulfides and diselenides due to the high melting points of other metal oxide or metal precursors^57^. Recently developed molten salt assisted approaches can also reduce the required growth temperature for WS_2_^57–59^, and has been extended to other TMDs that are not easily synthesized by CVD^57^. We envisage that the thin-film sulfurization approach can similarly provide a versatile method of synthesizing a broad range of TMDs, heterostructures or alloys based on the choice of precursors^30^. This approach may also lend itself to TMD nanostructure fabrication by pre-patterning the metal oxide precursor prior to sulfurization^60^.

Future experiments will look to further optimize and extend the procedure to fabricate other TMDs and heterostructures. Further considerations will also be given to the role of the substrate in the growth, and in tailoring electronic properties of the heterostructures. For example, by employing hydrogen intercalation to release the buffer layer from the SiC, the quasi-freestanding epitaxial graphene becomes p-type which in turn is expected to result in charge transfer and an interface dipole between WS_2_ and graphene layers^11,61^. Such approaches can be used to engineer ohmic contacts between TMD and graphene layers and optimize their photoconductive properties^11^.

## Conclusion

Van der Waals heterostructures of WS_2_ and epitaxial graphene have been synthesized by sulfurization of WO_3**−**x_ thin films deposited directly onto the epitaxial graphene/SiC substrate. The WS_2_ layer has a measured thickness of 1–3 layers and individual grains exhibit many rotational orientations with respect to the underlying graphene. As an alternative to conventional growth which requires growth temperatures in excess of 900 °C, WO_3**−**x_ thin-film sulfurization was demonstrated to produce WS_2_ at temperatures as low as 700 °C with no residual oxide. Below this temperature WS_2_ coexists with an intermediate WO_x_S_y_ compound. We anticipate that this process could be utilized to produce novel heterostructures of graphene with TMDs that cannot be synthesized by a conventional CVD method.

## Experimental Section

### Epitaxial graphene substrate preparation

Graphene was grown epitaxially on vicinal 4H-SiC(0001) substrates (4° off-axis towards $\left[ {11{ }\overline{2}0} \right]$, Norstel) by silicon sublimation in ultrahigh vacuum (UHV). The substrates were initially cleaned under ultrasonication for 15 min each in acetone, isopropanol and deionized water before introducing to UHV (base pressure ~ 1.0 × 10^−1^^0^ mbar). Samples were degassed at 600 °C until the pressure recovered to the 10^−1^^0^ mbar range, before annealing at 950 °C under Si flux to remove contaminants. The Si flux was generated by electron beam evaporation (EFM3, FOCUS GmbH) of a high purity Si rod (n-doped (P), ρ = 10–100 Ω cm, Hans Holm GmbH). For graphene growth, the samples were flash annealed to 1400–1500 °C while maintaining the Si flux. The sample temperature was measured using an optical pyrometer with an emissivity of 0.9. The temperature uncertainty is estimated to be ± 25 °C.

### WS_2_ synthesis

WS_2_/graphene/SiC heterostructures were prepared by sulfurization of WO_3**−**x_ thin films. The WO_3**−**x_ film was deposited from WO_3_ pellets (99.99%, Kurt J. Lesker) on the epitaxial graphene substrates by electron-beam physical vapor deposition (PVD75, Kurt J. Lesker) with a deposition rate of 0.5 Ås^−1^. The substrates were kept at room temperature and the chamber pressure was < 4.0 × 10^−6^ Torr during deposition. For WS_2_ synthesis, the samples were loaded into the downstream zone of the quartz tube furnace, and sulfur powder (1 g, 99.99%, Sigma Aldrich) was placed 20 cm upstream from the sample in a separately heated zone. Before the heating ramps were applied the tube was pumped to 10 Torr and purged with 600 sccm Ar for one hour. The Ar carrier gas flow rate was reduced to 400 sccm during sulfurization. The WO_3**−**x_/graphene/SiC sample was first heated to 150 °C for 20 min to remove adsorbed water, followed by a 60 min ramp to the sulfurization temperature, T_S,_ chosen in the range 650–800 °C. The sulfur powder was heated to 200 °C in a 15 min ramp, so that it reached the evaporation temperature at the same time as the sample reached the sulfurization temperature. The total sulfurization time was 20 min, which was sufficient to completely deplete the sulfur feedstock, and after sulfurization the furnace was allowed to cool naturally to room temperature.

### Material characterization

The sample morphology was characterized with atomic force microscopy (AFM) and scanning tunneling microscopy (STM). AFM images were acquired on an NT-MDT Solver microscope in tapping mode under ambient conditions. STM measurements were taken in UHV at room temperature using a VT-AFM/XA system (ScientaOmicron) with electrochemically etched W tips. The bias voltage was applied to the sample while the tip remained grounded. Before imaging, the samples were degassed at 400 °C for 30 min to desorb contaminants. Scanning probe microscopy data were analyzed using Gwyddion and SPIP^62,63^. X-ray photoemission spectroscopy (XPS) measurements were taken using a Kratos Axis Supra system with a monochromatic Al Kα source (1486.7 eV). Survey spectra were collected at 160 eV pass energy and high-resolution core level spectra were collected with a pass energy of 20 eV. The binding energy scale was calibrated by a rigid shift of the spectra to align the Si 2p_3/2_ core level of SiC to 101.6 eV. Ultraviolet photoemission spectroscopy (UPS) measurements were acquired on the same system using the HeI emission line (21.22 eV) and 10 eV analyzer pass energy. The binding energy scale was calibrated by aligning the Fermi level of an electrically contacted Au foil to 0 eV. XPS and UPS data were analyzed using CasaXPS^64^. Raman spectra were acquired using a Renishaw inVia Raman microscope equipped with a frequency doubled NdYAG laser (λ = 532 nm). The laser was focused on the sample with a 50 × objective lens giving a spot size of ~ 1 μm, and the laser power was limited to < 3.5 mW to prevent laser induced heating of the sample.

## Supplementary information

Supplementary file1
