# Supplementary material for: Synthesis and characterization of WS2/graphene/SiC van der Waals heterostructures via WO3−x thin film sulfurization
Source: Sci Rep. 2020 Oct 15;10:17334. doi: 10.1038/s41598-020-74024-w (PMC7567119; doi:10.1038/s41598-020-74024-w)
Supplement: Supplementary file 1 — Supplementary file1 [file 41598_2020_74024_MOESM1_ESM.pdf]

Supporting Information

Synthesis and Characterization of WS_2/Graphene/SiC van der Waals Heterostructures via WO_{3-x} Thin Film Sulfurization

Jonathan Bradford,[1,2] Mahnaz Shafiei,[3,4] Jennifer MacLeod,[1,4,5] and Nunzio Motta[1,4,5,*]

[1] *School of Chemistry and Physics, Queensland University of Technology (QUT), Brisbane, QLD, Australia*

[2] *School of Physics and Astronomy, University of Nottingham, Nottingham NG7 2RD, United Kingdom*

[3] *Faculty of Science, Engineering and Technology, Swinburne University of Technology, Hawthorn, VIC, Australia*

[4] *Institute for Future Environments, Queensland University of Technology (QUT), Brisbane, QLD, Australia*

[5] *Centre for Materials Science, Queensland University of Technology (QUT), Brisbane, QLD, Australia*

*Corresponding Author: n.motta@qut.edu.au

1. Characterization of the e-beam deposited WO_{3-x} layer

The XPS W 4f core level spectrum shown in Figure S1(a) shows a single state corresponding to tungsten oxide with W $4f_{7/2}$ at 35.8 eV and a spin-orbit peak separation of 2.1 eV. The high binding energy component at 41.3 eV arises from W $5p_{3/2}$ photoelectrons originating from the WO_3 layer. The corresponding peak in the O 1s core level (Figure S1(b)) appears at 530.7 eV in addition to components attributed to adventitious C-O and C=O species. Quantification of the peak areas corresponding to tungsten oxide indicate an O:W ratio of 2.5±0.3, and so the resulting layer is sub-stoichiometric WO_{3-x}. AFM topography images show a very flat surface with some small cracks as can be seen in Figure 2c. The root mean square (RMS) roughness of the WO_{3-x} is 0.72 nm, increasing slightly from 0.44 nm for the as-grown graphene/SiC surface. The crack located in the WO_{3-x} conveniently allows a direct measurement of the layer thickness. The line profile taken across the blue line in the topographic image as shown in Figure 2d indicates a true layer thickness of 10.8 nm (compared to the 10 nm nominal thickness).

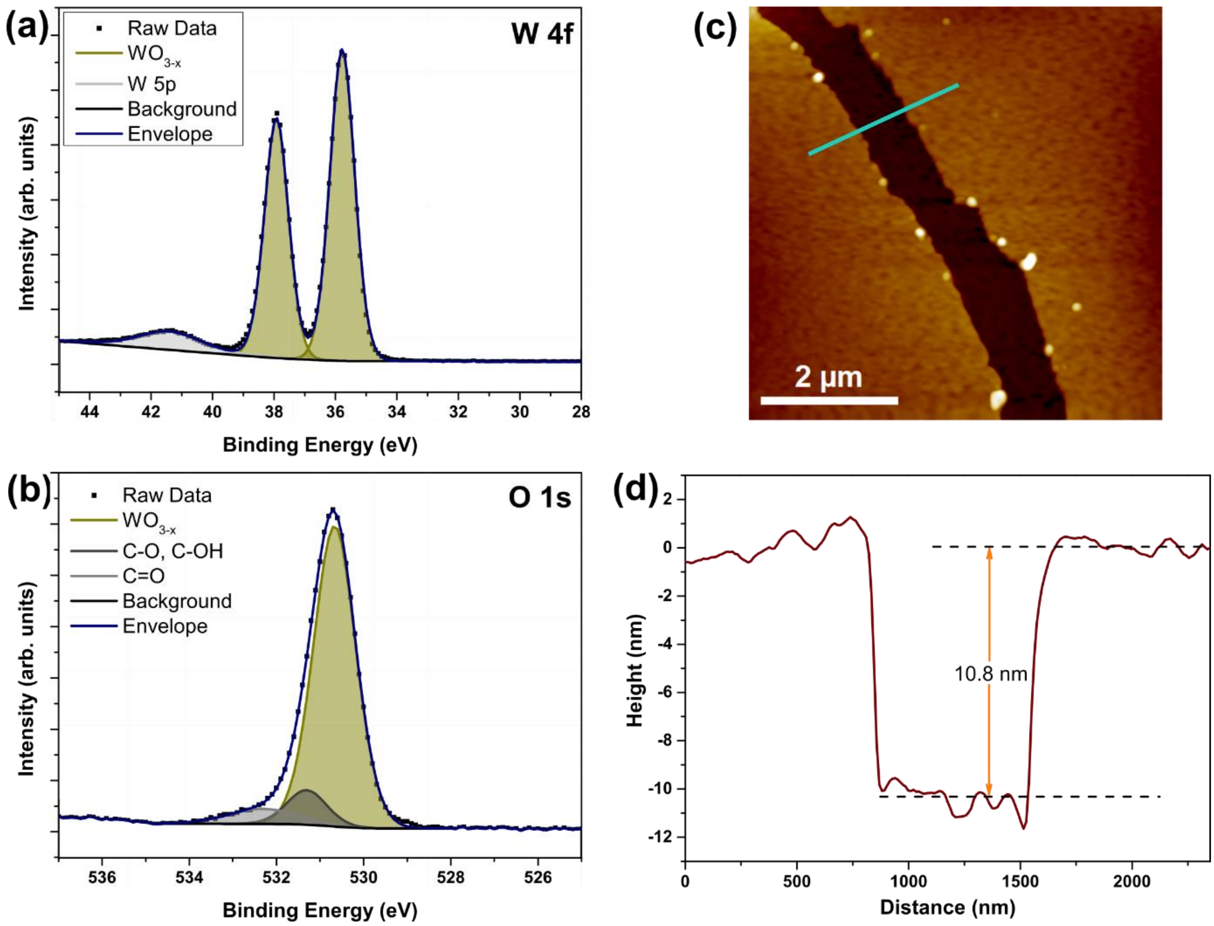

Figure S1. (a), and (b) XPS W 4f and O 1s core level spectra, respectively, of WO_{3-x}/graphene/SiC; (c) AFM topographic image of the WO_3 surface; and (d) Tip height line profile taken along the blue line in (c).

Figure S2 shows the Raman spectra of as-grown epitaxial graphene, and a sample after WO_3 deposition. All the Raman modes can be observed in both samples suggesting there is no signal arising from crystalline WO_3 phases. WO_3 exhibits Raman modes between 600 and 810 cm^{-1} depending on the phase.[1]

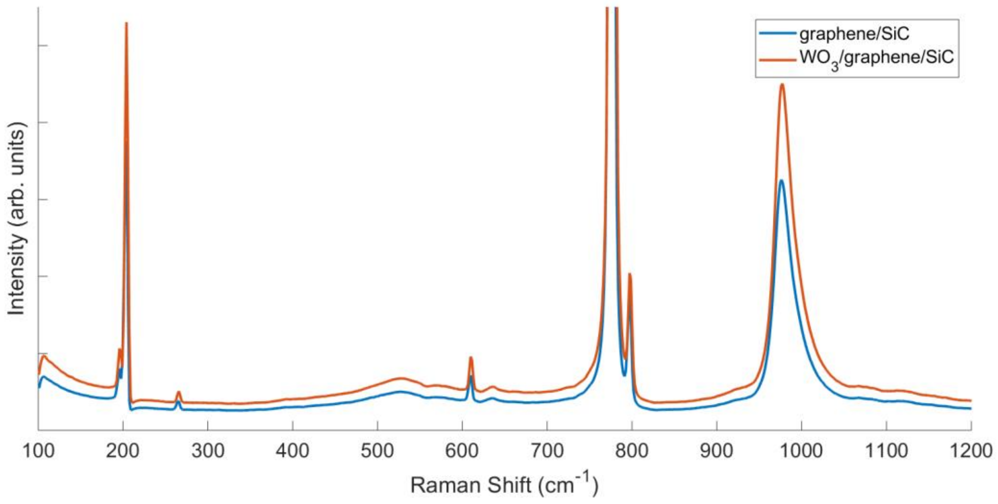

Figure S2. Comparison of the Raman spectra of graphene/SiC and WO_3/graphene/SiC. Both spectra are normalised to the SiC peak intensity.

2. High resolution XPS throughout the WS_2 growth process

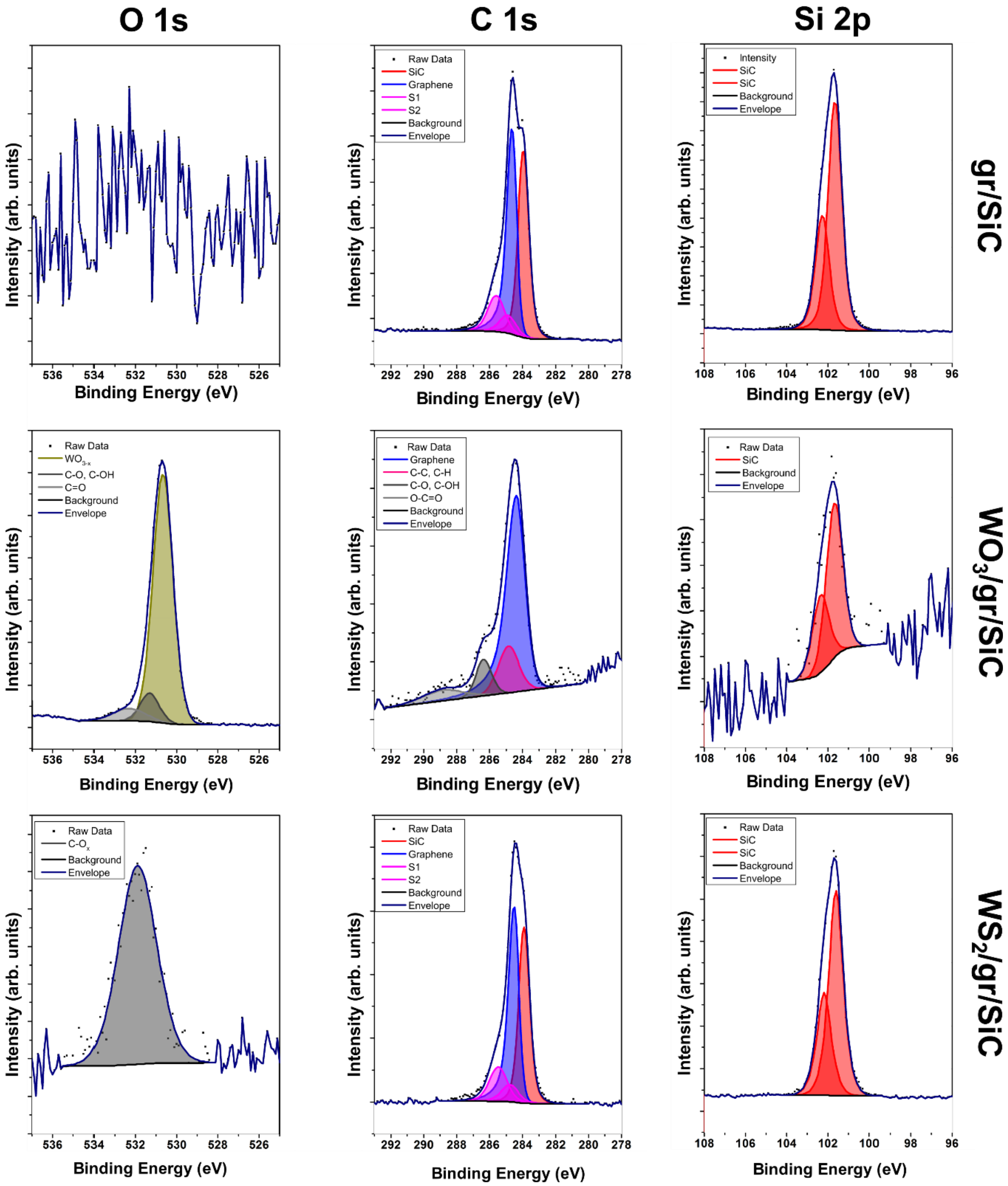

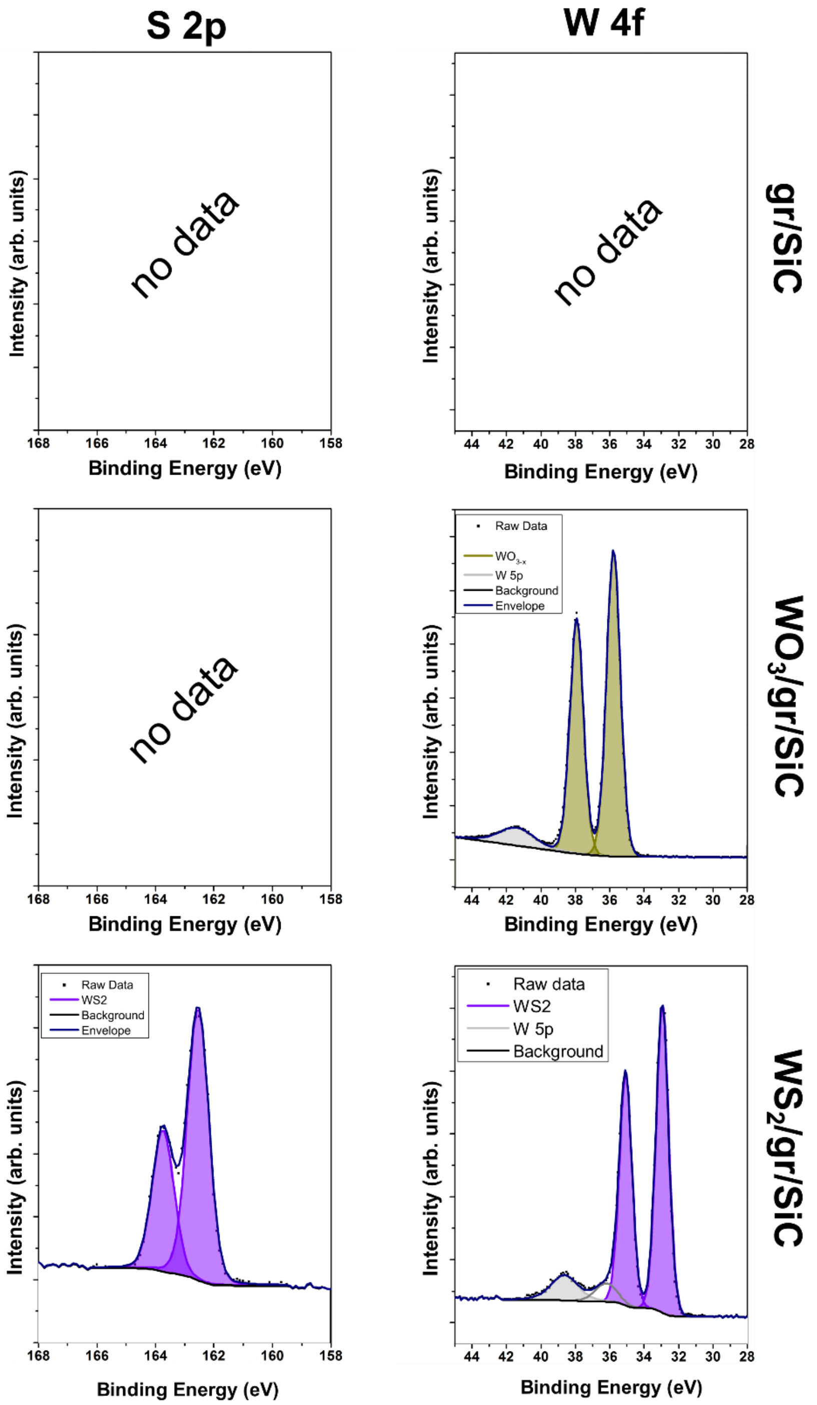

Figure S3. High resolution XPS core level spectra tracking the surface composition throughout the growth of WS_2 on epitaxial graphene/SiC by WO_{3-x} thin film sulfurization.

3. Additional STM Data and Discussion

Figure S4 shows additional STM data of the epitaxial graphene (EG) surface before and after WS_2 growth. The bare EG (Figure S4(a) and S4(b)) exhibits a series of steps separated by a few tens of nanometres, and the majority of step edges are oriented along or near to [1-100] and [10-10] directions of the SiC substrate. Figure S4(c) is a duplicate of the STM image in Figure 3(a) of the main text showing monolayer WS_2 domains on EG extending over the stepped EG surface. In this image the WS_2 is identified by domain boundaries which exhibit a step height of ~0.65 nm, corresponding to the thickness of a WS_2 layer, as shown in the line profiles in Figure S4(d). It is noted that the WS_2 edges have a greater apparent height than the centre of the WS_2 regions. This is attributed to a density of states effect since the image is acquired at a bias voltage within the WS_2 band gap, and edge states or defects result in a higher tunnelling conductance at the WS_2 edges.[1–4] The density of states effect is verified by the image in Figure S4(e) which is acquired at a positive bias voltage corresponding to empty WS_2 states, and thus the apparent thickness of the WS_2 layer is preserved across the whole domain (Figure S4(f)).

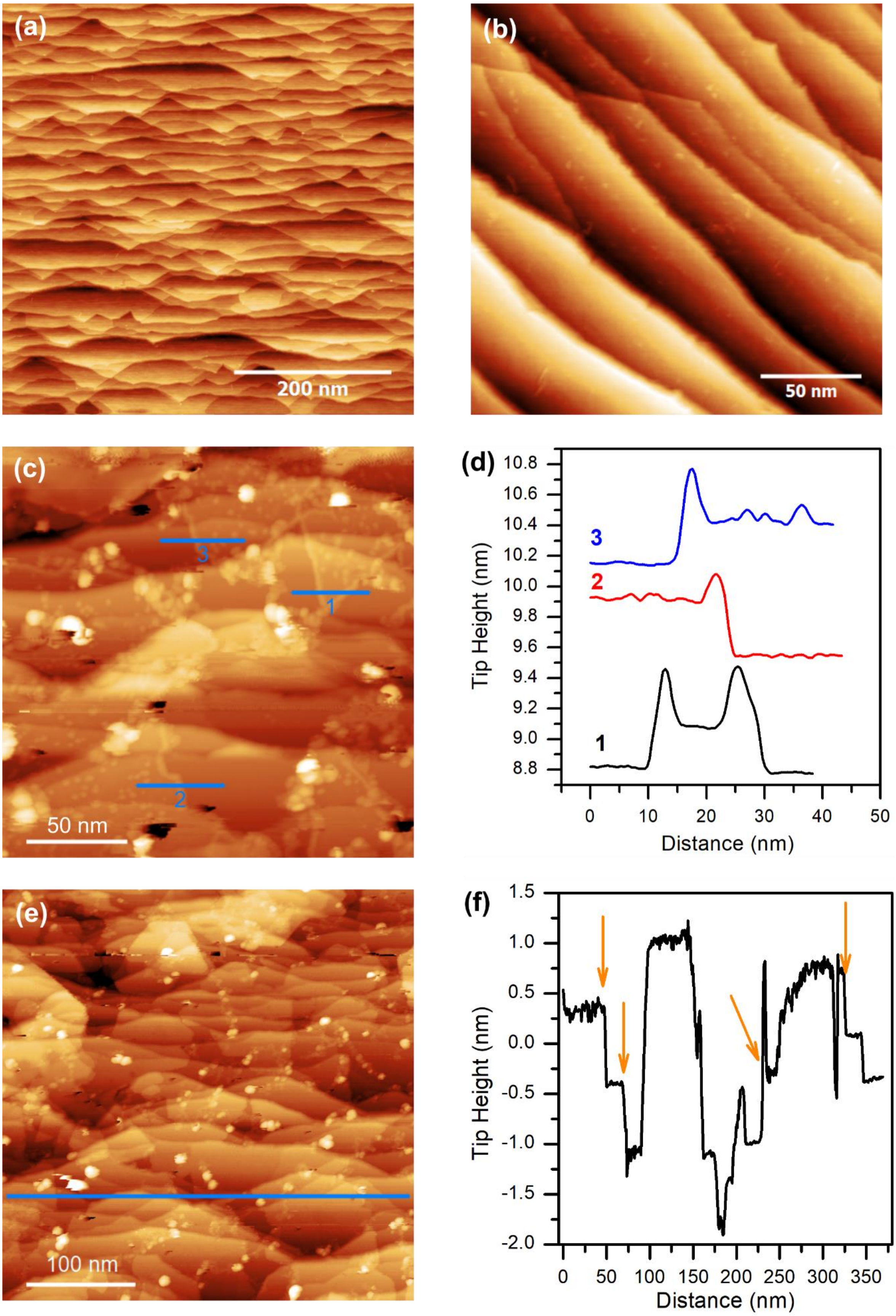

(a)
200 nm
(b)
50 nm
(c)
3
1
2
50 nm
(d)
3
2
1
Tip Height (nm)
Distance (nm)
(e)
100 nm
(f)
Tip Height (nm)
Distance (nm)

FigureS4. (a),(b) STM images of an epitaxial graphene surface prior to WS_2 growth. (c) STM image of monolayer WS_2 on epitaxial graphene (U = -1.50 V, I = 1.0 nA). The scale bar represents 50 nm. (d) Tip height line profiles extracts from left to right along the blue lines marked in (c). The lines are offset on the y-axis for clarity. (d) (350x 350) nm^2 STM image of WS_2 on epitaxial graphene showing varying numbers of WS_2 layers (U = 1.75 V, I = 1.0 nA). (e) Tip height line profiles extracts from left to right along the blue lines marked in (f). The orange arrows indicate steps with a height corresponding to monolayer WS_2.

4. WS_2/graphene/SiC SPA-LEED

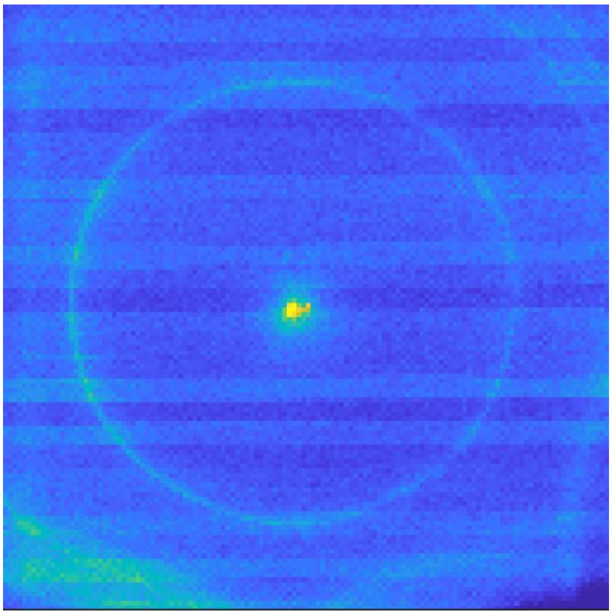

Figure S5. Spot-profile analysis low energy electron diffraction (SPA-LEED) pattern of the WS_2/graphene/SiC heterostructure. The pattern shows only a single diffraction ring originating from the WS_2 layer grown at 800 °C. This in indicative of a large variation in WS_2 grain orientation, and that there is no epitaxial relationship between the WS_2 and graphene layers.

5. Chemical Vapor Deposition of WS_2

WS_2 was grown by chemical vapor deposition from WO_3 powder and S powder precursors. 0.1 g of WO_3 powder was placed in an alumina crucible and placed in horizontal quartz tube furnace 2.5 cm upstream from the epitaxial graphene substrate. 1.5 grams of sulfur powder was placed outside the furnace and heated individually by a heating belt. The furnace was pumped to ~10 Torr and purged with 600 sccm Ar for 1 hr to create an inert environment. The Ar flow rate was then reduced to 200 sccm and the furnace was heated to 1100 °C to evaporate the WO_3 precursor. Once the furnace reached the growth temperature, the sulfur powder was evaporated at >200 °C. The WS_2 growth was maintained for 20 min before allowing the furnace to cool naturally under Ar flow.

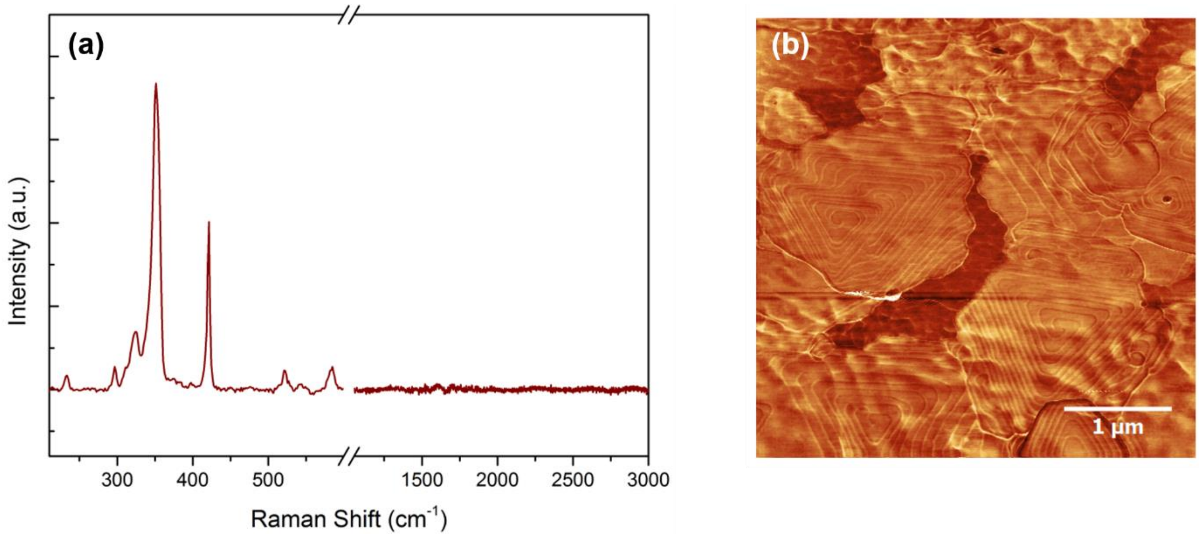

Figure S6. (a) Raman spectrum of CVD grown WS_2; (b) AFM phase image of WS_2 multilayers.

The Raman spectrum in Figure S6(a) shows the characteristic modes of WS_2, however the graphene bands are not visible. This is indicative that the graphene has degraded during the synthesis, presumably due to the presence of oxygen during the high temperature synthesis. The AFM phase image of the surface after WS_2 growth shown in

Fig. S6(b) shows multilayered WS_2 spirals structures like those previously observed by Sarma *et. al.*[2] and Fan *et. al.*[3]

References

[1] Boulova M and Lucazeau G 2002 Crystallite Nanosize Effect on the Structural Transitions of WO_3 Studied by Raman Spectroscopy *Journal of Solid State Chemistry* **167** 425-34

[2] Sarma P V, Patil P D, Barman P K, Kini R N and Shaijumon M M 2015 Controllable growth of few-layer spiral WS_2 *RSC Advances* **6** 376-82

[3] Fan X, Zhao Y, Zheng W, Li H, Wu X, Hu X, Zhang X, Zhu X, Zhang Q, Wang X, Yang B, Chen J, Jin S and Pan A 2018 Controllable Growth and Formation Mechanisms of Dislocated WS_2 Spirals *Nano Letters* **18** 3885-92
